# CoMAGC: a corpus with multi-faceted annotations of gene-cancer relations

**DOI:** 10.1186/1471-2105-14-323

**Published:** 2013-11-14

**Authors:** Hee-Jin Lee, Sang-Hyung Shim, Mi-Ryoung Song, Hyunju Lee, Jong C Park

**Affiliations:** 1 Department of Computer Science, KAIST, 291 Daehak-ro, Daejeon, Republic of Korea; 2 School of Life Sciences, Bioimaging Research Center and Cell Dynamics Research Center, Gwangju Institute of Science and Technology, 132 Cheomdan-gwagiro, Gwangju, Republic of Korea; 3 School of Information and Communications, Gwangju Institute of Science and Technology, 132 Cheomdan-gwagiro, Gwangju, Republic of Korea

## Abstract

**Background:**

In order to access the large amount of information in biomedical literature about genes implicated in various cancers both efficiently and accurately, the aid of text mining (TM) systems is invaluable. Current TM systems do target either gene-cancer relations or biological processes involving genes and cancers, but the former type produces information not comprehensive enough to explain how a gene affects a cancer, and the latter does not provide a concise summary of gene-cancer relations.

**Results:**

In this paper, we present a corpus for the development of TM systems that are specifically targeting gene-cancer relations but are still able to capture complex information in biomedical sentences. We describe CoMAGC, a corpus with multi-faceted annotations of gene-cancer relations. In CoMAGC, a piece of annotation is composed of four semantically orthogonal concepts that together express 1) how a gene changes, 2) how a cancer changes and 3) the causality between the gene and the cancer. The multi-faceted annotations are shown to have high inter-annotator agreement. In addition, we show that the annotations in CoMAGC allow us to infer the prospective roles of genes in cancers and to classify the genes into three classes according to the inferred roles. We encode the mapping between multi-faceted annotations and gene classes into 10 inference rules. The inference rules produce results with high accuracy as measured against human annotations. CoMAGC consists of 821 sentences on prostate, breast and ovarian cancers. Currently, we deal with changes in gene expression levels among other types of gene changes. The corpus is available at http://biopathway.org/CoMAGCunder the terms of the Creative Commons Attribution License ( http://creativecommons.org/licenses/by/2.0).

**Conclusions:**

The corpus will be an important resource for the development of advanced TM systems on gene-cancer relations.

## Background

For cancer research, it is essential to identify various genes that are involved in oncogenesis and to understand how the genes affect cancers. Since a large amount of information on such genes is contained in the literature, text mining (TM) has become invaluable [1-4].

TM systems that target genes associated either to cancer, or to other genetic diseases, are developed based on published corpora with annotations of gene-disease relations [5-10]. Some of these corpora contain simple binary relations where a gene and a disease form a positive pair if they are considered related to each other in any way [5,8]. Other corpora contain binary relations augmented with types or topics such as 'cause’ or 'expression’ [6,7,9,10]. Although TM systems based on such corpora may find disease-related genes efficiently, such pieces of information extracted by these systems are not yet comprehensive enough to explain how a gene affects a disease. There are also TM systems that target detailed information regarding genes and diseases, based on corpora with annotations of complex structures such as 'events’ [11-15]. For instance, the organizers of BioNLP Shared Task (ST) recently announced Infectious Diseases (ID) [14] and Cancer Genetics (CG) [15] tasks, and released corpora with annotations of pathological processes such as 'Carcinogenesis’ and anatomical entities such as 'Cell’ in addition to molecular processes and entities. However, such corpora do not still provide a concise summary of gene-disease relations, which may prove useful for efficient search for disease-related genes.

In this paper, we present the first steps towards TM systems that specifically identify gene-cancer relations but also capture more comprehensive information than other TM systems on gene-disease relations do. First, we describe CoMAGC, a corpus with multi-faceted annotations of gene-cancer relations. The multi-faceted annotation scheme of CoMAGC consists of four semantically orthogonal concepts that together express 1) change in gene property, 2) change in cancer property and 3) the causality between the gene and the cancer. In this regard, CoMAGC targets specifically the gene-cancer relations, but still captures complex information in biomedical sentences. Two biologists reviewed the multi-faceted annotation scheme, and the inter-annotator agreement (IAA) values are found quite high.

Second, we show that the information captured by the annotations in CoMAGC is comprehensive enough to facilitate both the inference of prospective roles of genes in cancers and the classification of genes into three classes according to the inferred roles. The three gene classes are 'oncogene’, 'tumor suppressor gene’ and 'biomarker’^a^. Such three-way classification of genes is useful in cancer research - we can distinguish the genes that are responsible for oncogenesis from other genes that are not, information essential for effective therapy design [16]. We encode the mapping between the multi-faceted annotations and the gene classes into 10 inference rules. The validity and applicability of the rules are confirmed by the two biologists, and the inference results show high accuracy when measured against human annotations of gene classes.

The corpus consists of 821 sentences collected from MEDLINE abstracts, and the sentences are about three different types of cancers, or prostate, breast and ovarian cancers. In the present work, we limit our attention to 'gene expressions’ among many other properties of genes. The proposed annotation scheme and the inference rules can be extended easily to incorporate change in other types of gene properties such as methylation and phosphorylation.

### Related work

While there are a few publicly available corpora on gene-disease relations [8,17-19], most of the current TM systems on gene-disease relations are developed with in-house corpora. Thus, we review the current TM systems focusing on the annotation schemes of their in-house corpora, or the definitions and formats of the gene-disease relations as used in each of the systems.

In the work by Craven and colleagues [5,17] and PolySearch [8], gene-disease relations are defined as simple binary relations. Craven and colleagues defined 'related’ pairs of genes and diseases as those registered as associated in the Online Mendelian Inheritance in Man (OMIM) database [20] and induced a hierarchical hidden Markov model (hHMM) to extract the related pairs. In PolySearch, a measure of relevancy, or the PolySearch Relevancy Index (PRI) score, is calculated upon word co-occurrences and pattern matching and assigned to each gene-disease pair.

In Gene Expression Text Miner (GETM) [18] and MeInfoText [10], gene-disease relations are formatted as also binary relations. However, in these two systems, the 'related’ gene-disease pair is defined in a narrower sense than those in the former two systems. GETM collects only the genes that are expressed in disease cells, and MeInfoText collects only the genes that show methylated status in cancer. Note that GETM was originally developed to identify mentions of gene expressions along with their anatomical locations, not restricted to disease cells. Variome [19], which relates mutated genes to diseases, is annotated with a scheme comparable to those employed by GETM and MeInfoText. However, the corpus deals with more diverse concepts and relations than only genes, diseases and the relation between the two, since it is developed to capture core concepts and relations relevant to cataloguing human genetic variation and its relationship to disease.

Chun and colleagues [6] format gene-disease relations as also binary relations but they propose to augment the binary relations with additional topics. First, they collected genes that are 'related’ to prostate cancer considering three perspectives: 'pathophysiology’, 'therapeutic significance’ and 'markers for prostate cancer’. Then, they assigned topics to the identified genes, where the topics include 'gene expression’, 'study description’, 'genetic variation’, 'epigenetics’, 'pharmacology’ and 'clinical marker’. Since the topics are mutually independent, one or more topics can be assigned to an established gene-prostate cancer relation.

Some systems employ binary relations with relation types, or typed binary relations, to represent gene-disease relations. Bundschus and colleagues [9] propose relation types such as 'altered expression’, 'genetic variation’, 'regulatory modification’, 'any’ and 'unrelated’, since they focus on how genes change in diseases. Masseroli and colleagues [7] label the relations with types 'ASSOCIATE_WITH’, 'PREDISPOSE’, 'CAUSE’ and negations of the three, focusing on the causality between genes and diseases. In both work, the authors build a network of genes and diseases using typed binary relations extracted by their systems to facilitate knowledge discovery. Pharmspresso [21] also employs typed binary relations. The system identifies relations between pharmacogenomic entities such as genes, diseases and drugs, and populates pharmacogenomic databases such as Pharmacogenomics Knowledge Base (PharmGKB) [22]. The relation types, which include 'Association’, 'Characterization’ and 'Effect’, are defined based on the Textpresso ontology [23].

DigSee [24] employs ternary relations among genes, cancers and biological events and identifies sentences describing that 'genes’ are involved in the development of 'cancer’ through 'biological events’. The system deals with several types of biological events such as 'Binding’, 'Gene expression’, 'Localization’, 'Phosphorylation’, 'Protein catabolism’ and 'Transcription’. The ternary relations in DigSee are comparable to those in the annotation scheme of CoMAGC, which is to represent ternary relations among genes, cancers and gene changes. Although DigSee deals with more diverse types of biological events when compared to the single type of gene change dealt with by CoMAGC, DigSee captures less detailed information than those captured by CoMAGC. For instance, DigSee captures neither whether the cancer progresses or regresses nor the details of biological events such as the directions of gene expression changes.

Apart from TM systems that specifically target gene-disease relations, there are systems that identify detailed information about genes and diseases. Early approaches include MedLEE [25] and GENIES [12], which employ a frame, a list that contains its type, value and possibly additional frames. In more recent work, the structure 'event’ has received much attention. An event consists of its type and participants, where one event can be a participant of another [11]. The structure has been used to represent a variety of biological processes, including molecular mechanisms of infectious diseases as proposed in the Infectious Diseases (ID) task of BioNLP Shared Task (ST) 2011 [14,26]. In the multi-level event extraction (MLEE) corpus [13], the structure is extended to incorporate anatomical entities and processes including disease cells and tissues. In the Cancer Genetics (CG) task of BioNLP ST 2013 [15], which aims to identify biological processes as related to the development and progression of cancer, the types of events are extended to include pathological and anatomical processes such as 'Carcinogenesis’ and 'Cell differentiation’.

**Figure 1:**
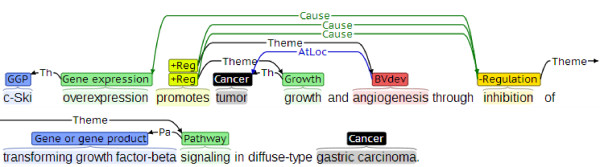
**Event annotations in the CG corpus.** We used the brat rapid annotation tool [27] for visualization of event annotations.

The two kinds of corpora, corpora with annotations of complex structures such as the CG corpus and corpora with annotations of gene-disease relations such as CoMAGC, both capture information regarding genes and diseases. However, they serve purposes quite different from each other. While the former supports development of TM systems that extract biological processes involved in cancers, the latter is suitable for the development of TM systems that search genes implicated in cancers in various ways. For instance, event annotations as shown in Figure 1, an excerpt from the CG corpus, capture detailed information about biological processes involving *c-Ski*, *transforming growth factor-beta* and *gastric cancer*. However, when only such event annotations are provided, one cannot readily identify *transforming growth factor-beta* and *c-Ski* as prospective suppressor and oncogene of *gastric cancer*, respectively. By contrast, CoMAGC aims to automate such identification of gene-cancer relations. Moreover, the two kinds of corpora are different in the characteristics of the annotated information, too. While corpora with complex structures are usually annotated with explicit mentions of biological processes, the annotations of gene-disease relations often contain information that is not explicitly stated but only implied in text. We expect that parallel annotations of the two kinds of information would produce interesting results. For instance, one may devise a method to automatically induce annotations for gene classification as in CoMAGC from the annotations of explicit mentions as in the CG corpus. The relevant study is left for future work.

## Results and discussion

### The annotation scheme

The four concepts that constitute the multi-faceted annotation scheme are Change in Gene Expression (CGE), Change in Cell State (CCS), Proposition Type (PT) and Initial Gene Expression level (IGE). Each of the four concepts is assigned with one of the pre-specified values to complete a piece of annotation. Table 1 shows the pre-specified values for each of the annotation concepts and the definitions of the respective values.

**Table 1:** Annotation concept values and their definitions

**Concept**	**Value**	**Definition**
CGE	increased	Expression level of the gene is increased.
	decreased	Expression level of the gene is decreased.
CCS	normal- >normal	The cell or tissue remains as normal after the change in the gene’s expression level.
	normal- >cancer	The cell or tissue acquires cancerous properties as the gene expression level changes; some cancerous properties of the cell or tissue are strengthened as the gene expression level changes.
	cancer- >cancer	There’s no change in the cancerous properties of the cell or tissue despite the change in the expression level of the gene.
	cancer- >normal	The cell or tissue loses some cancerous properties as the gene expression level changes; some cancerous properties of the cell or tissue are weakened as the gene expression level changes.
	unidentifiable	The information about whether or not the gene expression level change accompanies cell or tissue state change is not provided.
IGE	up-regulated	The initial gene expression level is higher than the expression level of the gene in the normal state.
	down-regulated	The initial gene expression level is lower than the expression level of the gene in the normal state.
	unchanged	The initial gene expression level is comparable to the expression level of the gene in the normal state.
	unidentifiable	The information about the initial gene expression level is not provided.
PT	observation	Cell or tissue change accompanied by the gene expression level change is reported as observed but the causality between the two is not claimed.
	causality	The causality between the gene expression level change and the cell or tissue change is claimed.

In the definitions, 'normal’ state of cells or tissues refers to the state in which the cells or tissues show no cancerous properties.

The four concepts together express, in a symbolic manner, information about a gene’s expression level change in a cell or tissue, hereafter *cell*, which accompanies change in cancerous properties of the cell. Here, cancerous properties of cells include self-sufficiency in growth signals, insensitivity to anti-growth signals, tissue invasion and metastasis, limitless replicative potential, sustained angiogenesis, evasion of apoptosis, tumor-promoting inflammation, destruction avoidance and deregulation of cellular energetics [28,29]. CGE captures whether the expression level of a gene is 'increased’ or 'decreased’ in a cell. CCS captures the way how the cell changes together with a gene expression level change. If the cell gets more cancerous following the gene expression change, we assign 'normal- >cancer’ to CCS. If the cell gets less cancerous, we assign 'cancer- >normal’, and if the cell exhibits no change in its cancerous properties, we assign 'cancer- >cancer’ or 'normal- >normal’. Lastly, when information about cell property change is not provided in the sentence, we assign 'unidentifiable’ to CCS. Note that we say that a cell becomes more cancerous when the cell acquires cancerous properties or when existing cancerous properties of the cell are strengthened. PT captures whether the causality between the gene expression change and the cell property change is claimed in the sentence or not, with the values 'causality’ and 'observation’.

The last concept, IGE, captures the initial expression level of a gene before the change in its expression level. We denote the initial gene expression level as 'up-regulated’, 'down-regulated’ or 'unchanged’, relative to the expression level of the gene in a normal cell that has no cancerous property. Here, we assume that the expression level of a gene in a normal cell is maintained at a certain level unless any external treatment is applied to the cell. We can use the values of IGE in combination with the values of CGE to deduce the causality between the gene expression change and the cell property change. Suppose that a gene is initially 'up-regulated’ in a cancer cell. If the cell becomes less cancerous as the expression level of the gene is 'decreased’, we can infer a causal relation between the gene expression level change and the change in cancerous properties of the cell. Such causality can also be inferred when IGE is 'down-regulated’ and CGE is 'increased’.

Note that the four annotation concepts are semantically orthogonal, in that the value of a concept can be identified not knowing the values of other concepts. Although 'causality’ PT is applicable only when CCS is either 'normal- >cancer’ or 'cancer- >normal’, we use the notion of orthogonality in a narrow sense, to mean that a concept can be defined and determined regardless of other concepts, and claim the orthogonality of the four annotation concepts among one another.

Table 2 shows example annotations. The unit of annotation is a mention of gene expression change that co-occurs with at least one cancer-related term in a sentence, where the annotators consult the sentence as the window of context during the annotation. Note that if CCS is 'unidentifiable’, identifying values of other concepts does not elaborate on the gene-cancer relation. Thus, we do not annotate PT and IGE when CCS is 'unidentifiable’. However, CGE is annotated regardless of CCS, because the concept is annotated first by using automatic means before annotating CCS.

**Table 2:** Example annotations with inferred gene classes

**Example sentence**	**CGE**	**CCS**	**IGE**	**PT**	**Gene class**
[*Cdc25B*]_*g*_ is frequently [*overexpressed*]_*e*_ in human [*prostate cancer*]_*c*_ tissues (29 of 30;97%). [PMID:12569365]	inc.	n- >c	unc.	obs.	Biomarker(by rule 7)
[*Downregulation*]_*e*_ of [*protein kinase C*]_*g*_ suppresses induction ofapoptosis in human [*prostatic carcinoma*]_*c*_ cells. [PMID:8612685]	dec.	n- >c	uni.	cau.	Tumor suppressor gene(by rule 4)
For example, some studies showed that CLU expression is increased in advanced stages of prostate cancer and that [*suppression*]_*e*_ of [*CLU*]_*g*_ [*expression*]_*e*_ sensitizes [*prostate cancer*]_*c*_ cells to chemotherapeutic drugs killing. [PMID:19879420]	dec.	c- >n	up.	cau.	Oncogene(by rule 3)

In the example sentences, gene names, cancer-related terms and the keywords for gene expression change are noted in square brackets and marked with subscripts 'g’, 'c’ and 'e’, respectively. The annotation concept values are abbreviated for brevity.

The multi-faceted annotation scheme of CoMAGC is originally designed to represent ternary relations among genes, cancers and gene changes. However, since we deal with only changes in expression levels among other types of gene changes, one may regard the current version of CoMAGC annotation scheme as essentially containing typed binary relations between genes and cancers. That is, one may regard the combination of the four annotation concept values such as (CGE:inc., CCS:n- >c, PT:cau., IGE:unc.) as relation types. We must extend the annotation scheme to incorporate various types of gene changes and thus exploit the full expressive power of its original design. Also, we note that the information annotated in CoMAGC is more complex than those that can be captured by multi-label sentence classification, since more than one annotation unit can be produced from a sentence when the sentence contains more than one mention of a gene expression change.

### Inference rules

We can classify genes into three classes by inferring their prospective roles in cancer progression or regression using the values of the four annotation concepts. The definitions of the three gene classes as used in this work are as follows: 

• Oncogene: a gene that causes cells to acquire cancerous properties, or a gene that strengthens cancerous properties of cells.

• Tumor suppressor gene: a gene that causes cells to lose cancerous properties, or a gene that weakens cancerous properties of cells.

• Biomarker: a gene that is not identified as an oncogene or a tumor suppressor gene but shows an altered expression level in cells that show cancerous properties when compared to the expression level of the gene in normal cells^b^.

We encode the mapping between the annotation concept values and the gene classes into 10 inference rules. Table 3 shows the rules. The rationale behind the rules is simple. First, if increased expression level of a gene accompanies cancer progression and there exists a causal relation between the change in gene expression and the cancer progression, then the gene is considered an 'oncogene’. Similarly, if decreased expression level of a gene accompanies cancer regression and there is causality, the gene is also considered an 'oncogene’. Second, if increased expression level of a gene accompanies cancer regression and the change in cancer is caused by the change in gene expression level, the gene is considered a 'tumor suppress gene’. Again, if decreased expression level of a gene accompanies cancer progression and there is causality, the gene is considered a 'tumor suppressor gene’. Third, if change in gene expression level accompanies change in cancer but there is no evidence of causality between the two, the gene is considered a 'biomarker’.

**Table 3:** Inference rules for gene classification

**Rule #**	**CGE**	**CCS**	**IGE**	**PT**	**Gene class**
1	increased	normal- >cancer	*	causality	oncogene
2	decreased	cancer- >normal	unidentifiable	causality	oncogene
3	decreased	cancer- >normal	up-regulated	*	oncogene
4	decreased	normal- >cancer	*	causality	tumor suppressor gene
5	increased	cancer- >normal	unidentifiable	causality	tumor suppressor gene
6	increased	cancer- >normal	down-regulated	*	tumor suppressor gene
7	*	normal- >cancer	*	observation	biomarker
8	*	cancer- >normal	unidentifiable	observation	biomarker
9	decreased	cancer- >cancer	up-regulated	observation	biomarker
10	increased	cancer- >cancer	down-regulated	observation	biomarker

The rules are not symmetric to each other. For instance, Rule 4 states that a gene can be classified as a 'tumor suppressor gene’ when a decreased expression level of the gene causes cancer progression, regardless of the IGE value. Rule 2 is about a similar case, where a decreased expression level of a gene causes cancer regression. However, Rule 2 requires IGE to be 'unidentifiable’ to infer the gene as an 'oncogene’. Rule 3, not Rule 2, covers the case where CGE is 'up-regulated’ and PT is 'causality’. Also, Rule 2 does not cover the cases where IGE is 'down-regulated’ or 'unchanged’ since we expect such cases to be rarely reported in biology articles (cf. Section on Inference rule development). Rules 5 and 8 are designed in a similar way. The asterisk denotes all the pre-defined values for the corresponding concept.

Rule 1 covers the cases where increase in expression level of a gene is reported as accompanying change in cell state for it to become more cancerous and the causality between the two is claimed in text. For such a case, the gene is inferred as an 'oncogene’. Rules 2, 4 and 5 are based on similar reasoning. In Rule 3, a cancer cell becomes less cancerous as the expression level of a gene decreases, and the causality between the change in gene expression level and the change in cell state is inferred from the values of IGE and CGE, which are 'up-regulated’ and 'decreased’, respectively. Thus, the gene is classified as an 'oncogene’. Rule 6 is designed similarly. In Rule 7, change in expression level of a gene is reported as accompanying change in cell state for it to become more cancerous without any claim of causality. Thus, the gene is classified as a 'biomarker’. Rule 8 is for similar cases. In Rule 9, change in expression level of a gene does not accompany any change in cell. However, the gene is classified as a 'biomarker’ because the gene initially shows 'up-regulated’ expression level in a cell that shows cancerous properties. Rule 10 is similar.

The 10 rules do not cover all the possible combinations of the annotation concept values, but they do cover all the plausible and biologically meaningful cases. The cases that are not covered by the rules are either highly unlikely to be reported in biomedical text or about genes that are not related to cancer. Thus, when there is no inference rule to apply, we classify the gene as not related to cancer.

Note that we do not claim that the gene classes inferred by the inference rules are definite. Rather, one should interpret the inferred gene classes and the corresponding annotation units as textual evidence that supports hypotheses on the prospective roles of the genes in cancers. To fully understand how a gene functions regarding cancers, one should collect many pieces of such textual evidence and conclude based on the collected evidence.

In this regard, one may relate the inference rules to functional gene annotation methods. In these methods, functions of genes would be predicted by utilizing various types of information such as biomedical text, nucleic acid sequence homology, gene expression profiles and protein domain [30]. Usually, gene functions are denoted as Gene Ontology (GO) terms. The inference rules proposed in this paper can be considered as a kind of functional gene annotation methods that utilize, in this case, biomedical text, since the three gene classes represent gene functions regarding cancers.

Table 2 shows the gene classes inferred from the example annotations.

### Corpus statistics

CoMAGC consists of 821 annotation units, where an annotation unit is a mention of gene expression change that co-occurs with at least one cancer-related term in a sentence. 310 of the annotation units co-occur with terms related to prostate cancer, 255 to breast cancer and 256 to ovarian cancer. The annotation units are derived from 408 PubMed abstracts, and contain 538 unique gene names. Table 4 shows the size of CoMAGC as compared to other publicly available corpora on genes and diseases.

**Table 4:** The sizes of corpora about genes and diseases

**Corpus**	**# documents**	**# words**	**Annotation**
CoMAGC	408	26177	821 sets of fourannotation concepts
Craven	1677	333845	829 gene-diseasepairs
PolsySearch	522	116380	341 gene-diseasepairs
GETM	150	38355	267 gene expression-anatomical locationpairs
MLEE	262	56588	6677 events
ID	30	153153	4150 events
CG	600	129878	17248 events

All the corpora contain PubMed abstracts, except for the ID corpus which contains full text documents. For the Craven and PolySearch corpora, we show the number of positive gene-disease pairs only.

Table 5 shows the distribution of annotation concept values in CoMAGC. For all the four annotation concepts, the distribution of the values is not uniform and sometimes extremely skewed. In particular, to CCS, only the values 'normal- >cancer’, 'cancer- >normal’ and 'unidentifiable’ are actually assigned. The absence of the two remaining values, 'normal- >normal’ and 'cancer- >cancer’, is due to the rarity of the negative results in biomedical literature as well as the fact that the 'change’ in cell properties is defined in a wide manner. In addition, for IGE, almost all the assigned values are either 'unchanged’ or 'unidentifiable’. Only two annotation units are annotated with the 'up-regulated’ IGE value, one of which is shown as the last example in Table 2. The value distribution of the concepts CGE and PT also exhibits dominance of a single value, 'increased’ and 'observation’, respectively.

**Table 5:** Distribution of the annotation concept values after adjudication

**Cancer type**	**CGE**		**CCS**			**IGE**			**PT**	
	**inc.**	**dec.**	**n- >c**	**c- >n**	**un.**	**up.**	**unc.**	**uni.**	**obs.**	**cau.**
Prostate	206(66%)	104(34%)	122(39%)	62(20%)	126(41%)	1(1%)	63(34%)	120(65%)	115(63%)	69(38%)
Breast	177(69%)	78(31%)	121(47%)	34(13%)	100(39%)	1(1%)	58(37%)	96(62%)	101(65%)	54(35%)
Ovarian	184(72%)	72(28%)	154(60%)	25(10%)	77(30%)	0(0%)	91(51%)	88(49%)	138(77%)	41(23%)
Total	567(69%)	254(31%)	397(48%)	121(15%)	303(37%)	2(0%)	212(41%)	304(59%)	354(68%)	164(32%)

The number of annotation units, to which each annotation concept value is assigned, is shown. The annotation concept values are abbreviated for brevity.

Although the four annotation concepts are semantically orthogonal to one another, the concepts show a strong correlation in their value distributions. Table 6 shows pairwise contingency matrices of the concepts. We can see from Table 6 that CCS is correlated strongly with all the other three concepts. First, CGE is four times more likely to be 'increased’ when CCS is 'normal- >cancer’ than when CCS is 'cancer- >normal’ (odds ratio of Table 6 (a)). Interestingly, however, when CCS is 'unidentifiable’, CGE and CCS are not strongly correlated to each other as indicated by the small residuals. Second, we can see from Table 6 (b) that 'unchanged’ IGE values are annotated only when CCS is 'normal- >cancer’. In addition, when CCS is 'cancer- >normal’, 98% of the IGE values are 'unidentifiable’ except for the two 'up-regulated’ values. Finally, PT is 6.5 times more likely to be 'observation’ when CCS is 'normal- >cancer’ than when CCS is 'cancer- >normal’ (odds ratio of Table 6 (c)). IGE and PT also show a correlation between each other. When PT is 'observation’, IGE is 7.8 times more likely to be 'unchanged’ than when PT is 'causality’ (odds ratio of Table 6 (d)).

**Table 6:** Correlation among annotation concepts

**(a) CGE - CCS (*p *** **<2.2** ***e *-16)**				
		**CCS**		
		n- >c	c- >n	un.
**CGE**	inc.	318 (2.65)	45 (-4.22)	204 (-0.36)
	dec.	79 (-3.95)	76 (6.30)	99 (0.54)
**(b) IGE - CCS ( *p *** **<2 *. *** **2 *e *-16)**				
		**CCS**		
		n- >c	c- >n	
**IGE**	up.	0 (-1.24)	2 (2.24)	
	unc.	212 (3.89)	0 (-7.04)	
	uni.	185 (-3.14)	119 (5.69)	
**(c) PT - CCS ( *p *** **<2 *. *** **2 *e *-16)**				
		**CCS**		
		n- >c	c- >n	
**PT**	ob.	311 (2.41)	43 (-4.36)	
	cau.	86 (-3.54)	78 (6.41)	
**(d) PT - IGE ( *p *** **<2 *. *** **2 *e *-16)**				
		**IGE**		
		up.	unc.	uni.
**PT**	ob.	0 (-1.17)	191 (3.83)	163 (-3.10)
	cau.	2 (1.72)	21 (-5.63)	141 (4.56)

Pairwise contingency matrices of the annotation concepts are shown. The analyses of correlation were performed by the *χ*^2^ test. Numbers in the parentheses are Pearson residuals. The annotation concept values are abbreviated for brevity.

In contrast, some pairs of concepts show weaker correlations. It seems that PT and CGE are independent of each other given the value of CCS; when conditioned to CCS, the null hypothesis of marginal independence between CGE and PT over IGE is not rejected by the *χ*^2^ test (*p* = 0.32). Similarly, IGE and CGE are likely to be independent of each other; the marginal independence between IGE and CGE over PT is not rejected by the *χ*^2^ test (*p* = 0.25), when CCS is conditioned to be 'normal- >cancer’. Note that we used only 517 annotation units with 'normal- >cancer’ or 'cancer- >normal’ CCS for correlation analyses regarding IGE or PT, because the values of IGE and PT are not annotated when CCS is 'unidentifiable’.

The correlations among the concepts suggest that there exist certain patterns in the sentences that report gene expression changes accompanying cancer changes. One may utilize such correlations or patterns to devise more effective TM methods. For instance, TM systems that identify strongly correlated concepts together at the same time may show better performance than the systems that identify each concept at a time.

Table 7 shows the distribution of applied inference rules. Rule 7 is applied most often, but Rules 6, 9 and 10 are not applied to any of the annotation units because there is no annotation unit that has 'down-regulated’ IGE, 'cancer- >cancer’ CCS or 'normal- >normal’ CCS. From 13%, 6% and 43% of the annotation units, oncogenes, tumor suppressor genes and biomarkers are inferred, respectively. For the remaining 37% of the annotation units, the genes are regarded as not related to cancer as there is no matching inference rule. Note that for all such annotation units with no matching inference rule, the CCS values are 'unidentifiable’. It is thus sensible to classify the genes as not related to cancer; if information about how a cell changes along with the gene expression change is not provided in the text, we can not make any inference about the gene’s role in cancer, but only conclude that the gene is not related to cancer since there is lack of evidence that the gene is implicated in cancer. Thus, it is confirmed from the distribution of the rule applications that the 10 inference rules effectively cover the cases that are likely to be reported in the literature and the cases that describe genes related to cancers.

**Table 7:** Distribution of applied inference rules

**Applied inference rule**	**Prostate cancer**	**Breast cancer**	**Ovarian cancer**	**Total**
1	21(7%)	26(10%)	22(9%)	69(8%)
2	21(7%)	13(5%)	9(4%)	43(5%)
3	1(0%)	1(0%)	0(0%)	2(0%)
4	5(2%)	6(2%)	6(2%)	17(2%)
5	21(7%)	8(3%)	4(2%)	33(4%)
6	0(0%)	0(0%)	0(0%)	0(0%)
7	96(31%)	89(35%)	126(49%)	311(38%)
8	19(6%)	12(5%)	12(5%)	43(5%)
9	0(0%)	0(0%)	0(0%)	0(0%)
10	0(0%)	0(0%)	0(0%)	0(0%)
No rule applied	126(41%)	100(39%)	77(30%)	303(37%)
Total	310(100%)	255(100%)	256(100%)	821(100%)

The number of annotation units to which each inference rule is applied is shown.

Table 8 shows genes that are inferred by the rules as oncogenes and tumor suppressor genes in CoMAGC. All the genes in Table 8 are registered as oncogenes or as tumor suppressor genes in biology databases such as UniprotKB [31], the Entrez Gene database at NCBI [32], OMIM [20] and TSGene [33] and in the cancer gene list by Vogelstein and colleagues [34]. Note that oncogenes and tumor suppressor genes in Entrez Gene and OMIM are retrieved via the Cancer Genes database [35]. We did not compare the biomarkers inferred by the rules to the biomarkers registered in biology databases, since the meaning of the term as used in biology databases is different from the meaning as used in this paper. In biology databases, a biomarker refers to a molecule that can be used as an indicator of a normal or abnormal process, or of a condition or disease [36]. In this context, oncogenes and tumor suppressor genes are often classified as biomarkers. Given the definition of biomarker in this paper, we regard the annotation units of CoMAGC as sufficient evidence to validate the classification of corresponding genes into biomarkers.

**Table 8:** Oncogenes and tumor suppressor genes in CoMAGC

**Cancer types**	**Oncogenes**	**Tumor suppressor genes**
Prostate cancer	FGF6^*u*^, PRKAR1A^*c*^, SPP1^*c*^, AR^*v*^, PTGS2^*c*^, CCND1^*vc*^, AKT1^*uvc*^, MMP2^*c*^, BCL2^*uvc*^	EAF2^*c*^, TFAP2A^*tc*^, WWOX^*utc*^, BRCA2^*utvc*^, PRKCD^*u*^, NKX3-1^*utc*^, MAPK8^*c*^, MAPKAPK5^*ut*^, NGFR^*t*^, ESR1^*c*^, CDKN1B^*c*^, NDRG1^*tc*^, BTG2^*tc*^, RBP1^*c*^, NFKBIA^*c*^, TGFB1^*tc*^
Breast cancer	TGFB1^*c*^, VEGFA^*c*^, ERBB2^*uvc*^, CCND1^*uvc*^, AGR2^*u*^, JUN^*uc*^, FOS^*uc*^, C11orf30^*c*^, AKT^*uc*^, PAK1^*c*^, FASN^*c*^, SRC^*uc*^	RBP1^*c*^, PFN1^*tc*^, PTEN^*utvc*^, IGFBP3^*tc*^, CDKN1A^*tc*^, ESR1^*c*^, TGFB1^*tc*^, MAPK14^*c*^
Ovarian cancer	PLAU^*c*^, TOP1^*u*^, VEGFA^*c*^, PAK1^*c*^, HGF^*c*^, MYC^*uvc*^, AKT2^*uc*^, ERBB2^*uvc*^	BRCA1^*utvc*^, BCL2^*vc*^, CAV1^*tc*^, CADM1^*utc*^, DUSP6^*tc*^

Genes marked with superscripts *u*, *c*, *t* and *v* are validated with UniprotKB [31], Cancer Genes database [35], TSGene [33] and the cancer gene list by Vogelstein and colleagues [34], respectively.

### Inter-Annotator Agreements (IAAs)

We annotated CoMAGC through four main annotation phases (cf. Table 9) and one supplementary phase, and revised annotation guidelines after each main annotation phase. Thus, we regard the IAA values obtained from the last of the main phases, or Phase 3, as the representative of the proposed annotation scheme and annotation guidelines.

**Table 9:** The four main annotation phases

**Annotation phase**	**# units**	**Cancer type**	**Data source**
Pilot	43	Prostate (43)	MEDLINE abstracts linked to DDPC
Phase 1	237	Prostate (237)	MEDLINE abstracts
Phase 2	451	Breast (225), ovarian (226)	MEDLINE abstracts
Phase 3	90	Prostate (30), breast (30), ovarian (30)	MEDLINE abstracts
Total	821	Prostate (310), breast (255), ovarian (256)	MEDLINE abstracts

Table 10 shows the IAA values obtained from each annotation phase as well as from the whole corpus. We measured IAAs in three different ways, using simple IAA, Cohen’s kappa and G-index [37]. Simple IAA is the proportion of agreed annotation units among all the annotation units. Cohen’s kappa is one of the most frequently used measures of agreement, which takes into account the agreement occurring by chance. However, Cohen’s kappa is known to devaluate the agreement rate when the distribution of the annotation categories is skewed [38]. G-index, which avoids such devaluation, is the same as Cohen’s kappa except that the chance agreement is calculated as the multiplicative inverse of the number of categories.

**Table 10:** IAA values

**Annotation phase**	**CGE**			**CCS**			**IGE**			**PT**		
	**Simple**	**Kappa**	**G**	**Simple**	**Kappa**	**G**	**Simple**	**Kappa**	**G**	**Simple**	**Kappa**	**G**
Pilot	1.00	1.00	1.00	0.73	0.34	0.66	1.00	1.00	1.00	0.83	0.63	0.67
Phase 1	1.00	0.99	0.99	0.81	0.71	0.76	0.95	0.48	0.93	0.90	0.64	0.81
Phase 2 - breast	0.99	0.98	0.98	0.75	0.60	0.69	0.96	0.00	0.95	0.84	0.65	0.69
Phase 2 - ovarian	1.00	0.99	0.99	0.80	0.63	0.75	1.00	N/A	1.00	0.88	0.62	0.75
Phase 2	0.99	0.98	0.99	0.78	0.62	0.72	0.98	0.00	0.97	0.86	0.64	0.72
Phase 3 - prostate	1.00	1.00	1.00	0.83	0.73	0.79	1.00	N/A	1.00	0.67	0.18	0.33
Phase 3 - breast	1.00	1.00	1.00	0.80	0.62	0.75	1.00	N/A	1.00	1.00	1.00	1.00
Phase 3 - ovarian	1.00	1.00	1.00	0.90	0.82	0.88	1.00	N/A	1.00	0.93	0.63	0.87
Overall	0.99	0.99	0.99	0.79	0.64	0.73	0.98	0.00	0.97	0.86	0.64	0.73

IAA values from the final phase show that adequate agreement among the annotators is achieved, except for the kappa on IGE, which is unobtainable or zero due to the extremely unbalanced distribution of IGE values. The overall IAA values, obtained from the whole corpus, suggest the internal consistency of CoMAGC. However, one should interpret the values as providing lower bounds, since the published version of CoMAGC contains only the annotation results after disagreement resolution, which is done after each annotation phase through annotator meetings.

#### Disagreements

We identify the following as the sources of the discrepancies among the annotators: simple mistakes, subjective readings of sentences, unrefined annotation scheme, and the use of background knowledge and inference during annotation. Discrepancies due to the latter two are greatly reduced in the later annotation phases, as we refined the annotation scheme and revised the guidelines through the phases.

Simple mistakes such as clicking misses and misunderstandings of sentences are the sole source of disagreements on CGE and IGE values. For the CGE value assignment, the annotators need to read only the keywords for gene expression changes that are provided as parts of annotation units (cf. Section on Annotation procedure procedure). No other information or inference is involved in the process. We regard such a simple nature of CGE annotation as the reason for the high IAA on CGE. All the small numbers of discrepancies on CGE are due to simple mistakes. In the case of IGE, the annotators agreed on 97% (87 out of 90) of the times to assign 'unidentifiable’^c^. Disagreements happened only for three annotation units, all due to simple misunderstanding of sentences by either of the annotators. Simple errors also take up 48% of the disagreements on PT values, and 69% on CCS values.

Discrepancies also arise when the same expression is interpreted differently between the annotators. Such subjective interpretation of the sentences contributes to most of the discrepancies on PT values, apart from simple errors.

##### 

**Example 1.** An [*increase*]_*e*_ in the activity of [*mitogen-activated protein kinase*]_*g*_ (MAPK) has been correlated with the progression of [*prostate cancer*]_*c*_ to advanced disease in humans. [PMID:15833840]

For the annotation unit as marked in Example 1, one annotator interpreted the word 'correlated’ as implying a causal relation and assigned 'causality’ to PT, but the other interpreted the word as having its literal meaning and assigned 'observation’ to PT. In fact, one annotator with background in natural language processing (NLP) always checked for explicit mentions of causality, while the other with background in biology interpreted expressions in a more context dependent manner. The one with biology background assigned 'causality’ 20% more often than the other with NLP background. Since we did not include any instructions on such subjectivity issues in our annotation guidelines, the IAA values on PT do not show significant improvement through the annotation phases.

Subjective readings induce disagreements on CSS values as well.

##### 

**Example 2.** These findings suggest that the quinazoline-based doxazosin mediates [*prostate cancer*]_*c*_ apoptosis by initially [*inducing*]_*e*_ the [*expression*]_*e*_ of [*TGF-beta1*]_*g*_ signalling effectors and subsequently I kappa B alpha. [PMID:12771931]

For the annotation unit as marked in Example 2, the annotator with biology background interpreted the verb 'mediates’ as conveying the meaning of 'positive regulation’ and assigned 'cancer- >normal’ to CCS. However, the other with NLP background interpreted the word as conveying only the meaning of 'regulation’ with no directionality and assigned 'unidentifiable’ to CCS. After annotator meeting, the CCS value of the annotation unit above was set to 'cancer- >normal’.

The IAA values on CCS from the first annotation phase, the pilot phase, are particularly low (cf. Table 10). This is greatly due to the unrefined annotation scheme at the pilot phase. The initial definition of CCS was only about cells, not including tissues. Also, the kind and the extent of cell changes encompassed by the 'normal- >cancer’ and 'cancer- >normal’ values were not specified.

##### 

**Example 3.** [*Increased expression*]_*e*_ of [*cyclin B1*]_*g*_ sensitizes [*prostate cancer*]_*c*_ cells to apoptosis induced by chemotherapy. [PMID:17513602]

For the annotation unit as shown in Example 3, one annotator assigned 'unidentifiable’ to CCS, claiming that if apoptosis occurs then a cell does not exist anymore. The other selected 'cancer- >cancer’, arguing that a cancer cell remains as cancer cell even after becoming more sensitive to chemotherapy. As the definitions of CCS values were refined to encompass tissues along with specification of cell changes, the CCS value of the annotation unit above is set to 'cancer- >normal’. With the refined definitions, the IAA values on CCS increased significantly. When compared to the values from the pilot phase, IAA values on CCS from Phase 1 show 11%, 108% and 15% increases in terms of simple, kappa and G index, respectively. In fact, from the annotation phases later than the pilot phase, discrepancies due to the unrefined annotation scheme were not observed at all.

After the annotation scheme was refined and revised, the main cause of disagreements on CCS was the use of background knowledge and inference during annotation, apart from simple errors.

##### 

**Example 4.** Treatment of the androgen-independent human [*prostate cancer*]_*c*_ cells [*PC-3*]_*c*_ with doxazosin resulted in a strong [*caspase-3*]_*g*_ [*activation*]_*e*_ within 24 h, whereas tamsulosin, a sulphonamide-based alpha 1-adrenoceptor antagonist, had no significant apoptotic effect against [*prostate cancer*]_*c*_ cells. [PMID:12771931]

One annotator assigned 'unidentifiable’ to CCS for the annotation unit in Example 4, as he did not find any explicitly stated piece of information on the effect of *caspase-3* on cancer cells. The other annotator, however, assigned 'cancer- >normal’ to CCS, as she inferred that the molecule has an apoptotic effect on cancer cells from the subordinate clause led by the word 'whereas’. After annotator meeting, the annotators set the CCS value for the annotation unit above to 'cancer- >normal’, and added an instruction that allows the inference using linguistic clues to the annotation guidelines. As we revised annotation guidelines adding new instructions on allowed and disallowed types of inference after each annotation phase, the disagreements on CCS due to different uses of background knowledge and inference among the annotators were greatly reduced. The effect of guideline revision is indicated by the high proportion of simple errors among the disagreement causes in the final phase. In Phase 3, 93% of the disagreements on CCS were due to simple errors, which is significantly increased when compared to the 66% in Phases 2 and 3.

### Inference rule validation

Although the inference rules stand to reason by themselves, we performed additional validation to confirm the applicability of the rules. We compared the gene classes inferred by the rules to the gene classes annotated by human annotators, where the annotators classified the genes directly as oncogenes, tumor suppressor genes and biomarkers, following the definitions of the terms. Two annotators performed such annotation for rule validation on 92 annotation units selected from CoMAGC. One of the two annotators participated in the main annotation of the four annotation concepts as well, while the other did not.

Table 11 shows the rule validation results. Agreement rates between the annotated gene classes and the inferred gene classes are shown. The micro-average agreement rate is 95%, and the macro-average agreement rate on the rules is 89%. There are five cases in which the inferred gene class is different from the annotated gene class, and we identified that none of such mismatching cases are due to errors in the rules themselves.

**Table 11:** Inference rule validation results

**Applied inference rules**	**Full match**	**One match**	**No match**	**Total**	**Agreement rate**
1	4	0	1	5	0.8
2	3	0	0	3	1
3	1	0	1	2	0.5
4	2	0	0	2	1
5	0	0	0	0	n/a
6	0	0	0	0	n/a
7	28	1	1	30	0.97
8	6	2	0	8	1
9	0	0	0	0	n/a
10	0	0	0	0	n/a
No rule applied	40	0	2	42	0.95
Total	82	3	5	92	0.95 (micro), 0.89 (macro)

A gene class inferred by an inference rule is compared with two gene classes annotated by each of the two annotators. When the three gene classes are all the same, we refer to the case as a *full match*. When the inferred gene class agrees with only one of the two annotated gene classes, we refer to the case as a *one match*. When the inferred gene class is different from both of the annotated gene classes, it is a *no match*. Agreement rate is calculated as the ratio of full match and one match among the total cases.

It seems that the annotators search for the evidence of gene-cancer association more actively, inferring more pieces of implicit information from the sentences, when they perform the main annotation of the four concepts than when they perform the validation annotation of the gene classes. Three of the five mismatching cases are due to such difference. Example 5 shows one of the three such cases.

#### 

**Example 5.** We conclude that 13q34 amplification may be of relevance in tumor progression of basal-like [*breast cancers*]_*c*_ by inducing [*overexpression*]_*e*_ of [*CUL4A*]_*g*_ and TFDP1, which are both important in cell cycle regulation. [PMID: 19995430]

For the annotation unit as marked in Example 5, CGE, CCS and PT are annotated as 'increased’, 'normal- >cancer’ and 'causality’, respectively. Hence, Rule 1 is applied and the gene *CUL4A* is inferred as an oncogene. In this case, the annotators inferred a causal relation between *CUL4A* overexpression and breast cancer progression from the expression 'by inducing’. However, for the validation annotation, the annotators classified the gene *CUL4A* as a biomarker, not inferring the causal relation.

At this point, it is difficult to answer the question of how much use of inference should be allowed for the task of gene classification. On the one hand, if no inference is employed, the gene classification will be based on more concrete evidence and become more accurate. On the other hand, if we classify genes with active use of inference, we can collect more genes that are likely to be oncogenes, tumor suppressor genes, and biomarkers. Thus, the appropriate extent of inference use depends on the research purpose at hand. We leave further analysis on this matter as future work.

In the remaining two of the five mismatching cases, the genes are inferred as not related to cancers since no inference rule is applied to the annotation units. However, the annotators classified the genes as biomarkers using information not about gene expression changes.

#### 

**Example 6.** In 2 cell lines with weak expression of TUBB3 protein ([*OVCAR-3*]_*c*_ and [*JHOC-8*]_*c*_), [*TUBB3*]_*g*_ [*induction*]_*e*_ was independently induced by treatment with 5-Aza-CdR ([*JHOC-8*]_*c*_) or PBA ([*OVCAR-3*]_*c*_), while neither agent markedly altered TUBB3 mRNA/protein expression in a strongly TUBB3-expressing cell line ([*JHOC-5*]_*c*_).

For the annotation unit as marked in Example 6, CCS is annotated as 'unidentifiable’ since how the cancer cell changes following the induction of *TUBB3* is not mentioned in the sentence. Accordingly, the gene *TUBB3* is inferred as not related to ovarian cancer. However, for validation annotation, the annotators classified the gene as a biomarker consulting the descriptions such as "weak expression of TUBB3 protein" and "strongly TUBB3-expressing cell line". Since the annotation scheme of CoMAGC and the proposed inference rules deal with textual descriptions about only gene expression changes, other descriptions of oncogenes, tumor suppressor genes and biomarkers cannot be identified when the description does not include explicit mentions of gene expression changes.

### Discussion

Although CoMAGC captures more comprehensive information than other corpora on gene-disease relations, annotations in CoMAGC may still be not informative enough for some tasks of cancer research. In particular, since the pre-specified values of the annotation concepts are defined at an abstract level, we cannot capture concrete features of gene-cancer relations with the proposed annotation scheme. For instance, the values for CCS are about only the directions of cell changes and are not about the exact cell properties that are altered. Thus, the annotations in CoMAGC would be of only limited help for such tasks as identification of genes that contribute to specific stages of oncogenesis such as the onset of malignant growth or metastasis. Therefore, one may want to redefine the pre-specified values of the annotation concepts to incorporate concrete features of gene-cancer relations into the annotation scheme. For such reorganization of annotation scheme, we anticipate that one can exploit the semantic orthogonality of the four annotation concepts. The pre-specified values of an annotation concept can be redefined neither considering nor affecting other concepts, and one needs to re-annotate only the reorganized concept on top of the existing corpus.

CoMAGC currently contains information regarding change in expression levels of genes among other properties of genes. On the one hand, we expect that such information about gene expression changes will be particularly useful for cancer research. In particular, the information can be used for the research on epi-driver genes, or genes that are expressed aberrantly in cancers in a fashion that confers a selective growth [34]. Since epi-driver genes are expected to explain a large portion of genetic mechanisms of oncogenesis that is not yet fully understood, further research on epi-driver genes is essential [34].

On the other hand, one may want to extend the annotation scheme to incorporate new types of gene alteration, because it is also important to identify whether genes show other types of alteration such as methylation in cancers or not. For such extension, we can use CCS and PT without any adjustment but should define two new concepts that correspond to IGE and CGE, respectively, as the two are about gene expression levels. For instance, for the mention of gene methylation marked in Example 7^d^, we can assign 'normal- >cancer’ to CCS and 'observation’ to PT, using the definitions and the pre-specified values of the two concepts as described in this paper.

#### 

**Example 7.** [*Hypermethylation*]_*e*_ of [*RASSF1A*]_*g*_ gene was found in circulating tumor-specific DNA in 43.1% of patients (22 out of 51 cases) with [*ovarian cancers*]_*c*_ (P <0.05). [PMID: 16545186]

Also, recall that the IGE values were 'unchanged’ or 'unidentifiable’ 99% of the time. Despite the fact that IGE is originally included in the annotation scheme for the inference of causality, there are only 2 annotation units whose IGE values are used in such way. Nevertheless, we expect to gain more IGE values that are neither 'unidentifiable’ nor 'unchanged’ when the window of context wider than a sentence is used. In fact, for the six annotation units with 'unidentifiable’ IGE, which are randomly selected from the pilot phase, we identified four 'up-regulated’ or 'down-regulated’ IGE values when we consulted an abstract as the window of context. Thus, it would be an interesting future work to re-annotate IGE values with a wider window of context than a sentence and compare the gene classification results to the current gene classification results.

Lastly, we must discuss the high IAA shown by the CoMAGC annotations. Attaining high quality annotations under the CoMAGC annotation scheme may seem to be a difficult task, since we allowed the annotators to perform inference during the annotation process, despite the previous report by Kim and colleagues [11] such that the restriction of annotations to actual expressions in text is a key device to reduce annotator discrepancies. While we did control the usage of inference with full annotation guidelines (cf. Section on Annotation guidelines), we may attribute the high IAA also to the following two factors. First, the CoMAGC annotation task has different characteristics from that of the annotation task performed by Kim and colleagues [11] or by other work that employs "events". For event annotation, the annotators should identify events from each abstract. In particular, they should specify keyword for each event as well as the type and the arguments of the event, not knowing the exact number of events in each abstract in advance. On the other hand, for CoMAGC annotation, the annotators need only to choose appropriate values for the four annotation concepts for each annotation unit. Such a simpler setting of the CoMAGC annotation task makes it easier to control the degree of inference during annotation. Second, the two annotators who performed the main annotation of CoMAGC had a largely similar amount of cancer knowledge, since neither was an oncologist. We anticipate that, if one of the annotators had a strong expertise in oncology, we would have needed a much longer list of annotation guideline instructions, having more difficulty in achieving good IAA. In this regard, we suggest that those who plan to construct a corpus allowing inference during annotation should deliberate on the degree of simplicity of the annotation task and the level of annotator expertise before performing the actual annotation.

## Methods

### Annotation procedure

We annotated CoMAGC in five phases: four main phases and one supplementary phase. In the main phases, two annotators performed the annotation, measured IAAs and revised annotation guidelines after the completion of each phase. CGE and CCS are annotated for all the annotation units, but PT and IGE are annotated only when CCS is 'normal- >cancer’ and 'cancer- >normal’, respectively. We assumed that PT should be 'causality’ when CCS is 'cancer- >normal’ and that IGE should be 'unchanged’ when CCS is 'normal- >cancer’. Although such assumptions seemed valid during the preliminary data analysis with small amount of data, we later discovered many cases that counter our assumptions when we re-examined a larger number of sentences after the completion of the main phases. Thus, we decided to perform additional annotations and added a new annotation phase. In this supplementary phase, one annotator who participated in the main phases annotated PT and IGE that are omitted during the main phases. Although we did not measure IAAs for the annotations in the supplementary phase, we claim that the quality of the annotations in the supplementary phase is comparable to the quality of the main phases since the annotators were trained during the main phases and consulted the final version of the annotation guidelines.

Table 9 shows the characteristics of the four main annotation phases. Starting from a small number of sentences with a focused domain, we gradually increased the number of sentences and broadened the domain for each annotation phase. In the pilot phase, we tested our annotation scheme on 43 annotation units extracted from MEDLINE abstracts registered to DDPC, or Dragon Database of Genes Implicated in Prostate Cancer [39]. By using only the abstracts registered to DDPC, we confined the data source to the abstracts that are about genes related to prostate cancer. In Phase 1, we increased the number of annotation units and extended the data source to the whole MEDLINE. In Phase 2, we included previously unseen cancer types, breast cancer and ovarian cancer. Finally, in Phase 3, we annotated an equal number of annotation units for each of the three cancer types to measure the final IAAs.

After the annotation for each phase is completed, the annotators held meetings to resolve the discrepancies and to revise the guidelines. The annotation guidelines were revised by adding additional guidelines or fine-tuning existing guidelines with more details. Thus, we didn’t need to revise the annotations done in the earlier phases.

Each annotation unit was presented to the annotators as a sentence with markings for a gene name, keywords for gene expression change and cancer-related terms. The annotators read the sentence with markings and selected proper values for the four annotation concepts using the drop-down lists of MS Excel files.

One of the two annotators who performed the annotation in the main phases is a graduate student majoring in developmental biology, and the other is a graduate student majoring in NLP in biomedical domain. Although the two annotators are not oncologists, they were able to understand the sentences about gene expression change and cancers to the extent sufficient enough for the annotation task. Also, the two, having different backgrounds from each other, were able to establish a balanced perspective between biology-oriented and NLP-oriented interpretations of the sentences.

#### Annotation guidelines

The proposed annotation scheme does not require annotations to be anchored on specific keywords or expressions. Rather, the annotation concept values are selected considering all the information conveyed in a sentence, which is the window of context. Hence, the annotators were allowed to perform inference during annotation. However, since allowing unrestricted inference would undermine the annotation quality and lower the IAA rates, they specified the spectrum of inferences that are allowed or disallowed as annotation guidelines. Table 12 shows the guidelines. Note that the guidelines are not about specific cancer types but about cancers in general, thus they can be applied to annotation regarding any type of cancers. The reader is referred to Additional file 1 for detailed explanations on the instructions in Table 12.

**Table 12:** Instructions on the allowed or disallowed inference types during annotation

**#**	**Instruction**
1	Annotators can interpret the sentences and annotate concepts in a 'conventional way’, in which the sentences would usually be interpreted by human readers.
2	Annotators can infer information using their prior knowledge about properties of cancer cells when the sentence is about comparison of two different cancer cells of the same cancer type.
3	Annotators can infer information utilizing linguistic clues.
4	Annotators should not infer information using their prior knowledge about the functions of genes.
5	Annotators should not infer the CCS value from the information about patients’ survival rates because progression of cancer cells is not the sole factor that contributes to patient survival or death.
6	Annotators need not consider the certainty level of propositions.

See Additional file 1 for more details on the instructions.

### Data collection and pre-processing

The unit of annotation in CoMAGC is a mention of gene expression change that co-occurs with at least one cancer-related term in a sentence. In this section, we describe the process to prepare the annotation units.

We first collected sentences about cancers from the MEDLINE. We downloaded abstracts via PubMed with queries 'prostate cancer’, 'breast cancer’ and 'ovarian cancer’, and randomly selected around 2,000 abstracts for each of the three cancer types and segmented them into sentences. We then selected only the sentences that contain cancer-related terms.

Cancer-related terms are identified by dictionary-based longest matching with an in-house cancer dictionary. The dictionary consists of cancer names retrieved from UMLS Metathesaurus [40], cancer cell line names collected from review papers and databases [41-51], and the lexicographic variants of the cancer names and the cell line names. To collect cancer names from UMLS Metathesaurus, we searched the Metathesaurus for concepts (CUIs) of 'Neoplastic Process (T191)’ semantic type using queries 'prostat*’, 'ovar*’ and 'breast*’, and then collected strings (SUIs) that correspond to the retrieved concepts. The lexicographic variants are produced using the Lexical Tools [52].

After collecting the sentences, we used text mining tools to identify gene names and mentions of gene expression changes from the sentences. We first tokenized, POS tagged and parsed the sentences using the Charniak-Johnson parser [53] with a biomedical parsing model [54]. The phrase structures produced by the parser are converted into dependency structures by the Stanford conversion tool [55] with the output option 'collapsed dependencies with propagation of conjunct dependencies’. We then used BANNER [56], trained on BioCreative 2 gene mention training set [57], to identify DNA, RNA and protein names, and Turku Event Extraction system (TEES) [58] to identify mentions of gene expression changes. Among all the mentions of molecular events identified by TEES, we selected only the mentions of 'Positive_regulation’ or 'Negative_regulation’ type events whose 'theme’ arguments are 'Expression’ type events or 'Protein’ type entities. Note that event mentions may refer to the regulation of gene functions as well as the regulation of gene expressions when the 'theme’ arguments of the events are 'Protein’ type entities. We did not differentiate the two cases since the proposed annotation scheme and the inference rules can be applied to both cases.

Finally, we manually validated the automatically identified mentions to produce confirmed annotation units. An automatically identified DNA, RNA or protein name is confirmed as correct if the text span denotes a specific sequence or a group of the molecules, excluding an entity too generic such as 'protein’ or 'promoter’. To examine the correctness of gene expression change mentions, we consulted the annotation guidelines of the GENIA event corpus [11], since TEES was trained on the BioNLP ST 2009 dataset [59] which is based on the GENIA event corpus. We also discarded the annotation units produced from the sentences that describe hypotheses or study purposes since scope ambiguity of hedging expressions brought significant inter-annotator discrepancies that are out of the scope of this study.

Although we employed manual work for some parts of the annotation unit production process, i.e., 1) cancer dictionary construction and 2) elimination of sentences that describe hypotheses or study purposes (*hypothesis sentences*), there are ways to automate these manually processed parts. First, instead of dictionary matching, one may use existing tools such as MetaMap [60] for cancer-related term identification. Also, when one targets a few types of cancers rather than all types of cancers, manual construction of dictionaries can still be an option. Second, we expect that one can easily build a system that automatically filters out hypothesis sentences based on previous work [61,62], which provides an annotated dataset along with algorithms with good performance. We also manually validated automatically identified gene names and gene expression change mentions in order to prevent false positive mentions from entering the corpus. Although errors in TM systems may be inevitable, one may devise post-processing filtering methods to minimize the effect of false positive errors. We leave the implementation of these suggestions for future work.

### Inference rule development

We first developed the basic idea behind the rules, which is explained in Section on *Inference rules*, by reading abstracts about genes that show altered expression levels in prostate cancer. The abstracts are retrieved via DDPC [39], a database about genes implicated in prostate cancer. Two biologists confirmed the basic idea after they examined the annotations and the inferred gene classes from the pilot phase. Then, we listed all the possible combinations of annotation concept values as shown in Additional file 2 and discarded the ones that are not logically possible. To the remaining combinations of annotation concept values, we assigned gene classes to be inferred. When IGE is 'unidentifiable’, we considered all the gene classes that would be inferred if IGE was assigned a different value such as 'up-regulated’, 'down-regulated’ or 'unchanged’. Among the possible gene classes, we selected the gene class that represents the weakest gene-cancer relation and assigned the class to the value combination with 'unidentifiable’ IGE. Here, 'not related to cancer’ represents the weakest relation while 'oncogene’ and 'tumor suppressor gene’ represent the strongest. For example, suppose that the values of CGE, CCS, PT and IGE are 'increased’, 'cancer- >normal’, 'observation’ and 'unidentifiable’, respectively. If IGE was 'down-regulated’, 'tumor suppressor gene’ would be inferred because causality between the gene and the cancer can be deduced from the values of IGE and CGE. If IGE was 'up-regulated’ or 'unchanged’, 'biomarker’ would be inferred because change in gene expression accompanies change in cell state but there is no evidence of causality. As a result, we assign 'biomarker’ to the example value combination above, since 'biomarker’ represents a weaker gene-cancer relation than 'tumor suppressor gene’. Finally, we discarded the combinations that we expect are rarely reported in biomedical articles, and summarized the remaining cases as 10 rules shown in Table 3.

### Annotation for inference rule validation

For the annotation of gene classes, which is to validate inference rules, we used 92 annotation units selected from CoMAGC. We selected all the 90 annotation units in Phase 3, one from Phase 1 and another from Phase 2. The two annotation units from Phases 1 and 2 are included to validate inference rule 3, which is not applied to any of the annotation units in Phase 3. Each annotation unit is presented to the annotators as a sentence marked with only the gene name and cancer-related terms, but without markings of the gene expression change mentions.

Two annotators performed the validation annotation. One of the two annotators, who participated also in the main annotation, is a graduate student majoring in natural language processing in biomedical domain. The other annotator, who did not participate in the main annotation, has a Ph.D in computer science who works on bioinformatics. The annotators consulted the annotation guidelines developed during the main annotation to square the level of inference usage with the level at the main annotation. The IAA values for the validation annotation are 0.72, 0.52 and 0.62 in terms of simple agreement, Cohen’s kappa and G-index, respectively. The IAA values are relatively low since one of the annotators who did not participate in the main annotation were unfamiliar to the guideline instructions. The annotators held a meeting to resolve the discrepancies due to such unfamiliarity and produced a revised version of gene class annotations, in which the annotators agreed on 89 gene classes among 92. We used such a revised version of gene class annotations for inference rule validation.

## Conclusions

In this paper, we present CoMAGC, a corpus with multi-faceted annotations of gene-cancer relations. CoMAGC is developed in order to support development of advanced TM systems on gene-cancer relations, which extract more comprehensive information than those extracted by current TM systems on gene-cancer relations. The multi-faceted annotation scheme of CoMAGC is a novel structured format that can express 1) how a gene changes, 2) how the cancer changes and 3) the causality between the gene and the cancer. The multi-faceted annotations have high agreement among the annotators. In addition, we showed that the information represented by the proposed annotation scheme is informative to the extent that it allows us to classify genes into oncogenes, tumor suppressor genes and biomarkers, according to the prospective roles of the genes in cancers. The 10 inference rules that describe the mapping from the annotation results to the gene classes produce results with high accuracy when measured against human annotations of gene classes. We anticipate that many TM systems will be developed with CoMAGC and utilized in various ways for cancer research.

## Endnotes

^a^ Despite the potential controversy, we adopt the definitions of these terms in our context based on their wide uses.

^b^ 'Biomarker’ as used in this paper indicates not only those genes that affect cancers but also those that are affected by cancers.

^c^ Note that IGE is annotated only when CCS is 'cancer- >normal’ during the main annotation phases (cf. Section on Annotation procedure) and that 98% of the IGE values are 'unidentifiable’ when CCS is 'cancer- >normal’ (cf. Table 6).

^d^ In Example 7, gene name, cancer-related term and keyword for methylation are marked similarly as in other examples.

## Competing interests

The authors declare that they have no competing interests.

## Authors’ contributions

JCP and HL initiated and designed the research. HL and HJL developed the annotation scheme and the inference rules, which are reviewed by MRS and SHS. SHS and HJL performed the main annotation of annotation concepts and developed annotation guidelines. HL and HJL performed the validation annotation for gene classes. JCP supervised all steps of the research. All authors read and approved the final manuscript.

## Supplementary Material

Additional file 1 **Corpus annotation guidelines.** A '.doc’ file that contains the definitions of annotation concept values, example annotations and instructions on allowed or disallowed types of inference during annotation. Click here for file

Additional file 2 **Complete combination of annotation concept values.** A '.xlsx’ file that lists the complete combinations of annotation concept values. The file also describes detailed reasoning processes behind the inference rules and explains the reason why some combinations are excluded from Table 2. Click here for file

## References

[B1] Rebholz-Schuhmann D Oellrich A Hoehndorf R Text-mining solutions for biomedical research: enabling integrative biology Nat Rev Genet 2012 13 12 829 839 10.1038/nrg3337 23150036

[B2] Hirschman L Burns GAPC Krallinger M Arighi C Cohen KB Valencia A Wu CH Chatr-Aryamontri A Dowell KG Huala E Lourenco A Nash R Veuthey A Wiegers T Winter AG Text mining for the biocuration workflow Database 2012, **2012**. doi:10.1093/database/bas020, [ http://database.oxfordjournals.org/content/2012/bas020.full] 10.1093/database/bas020 PMC3328793 22513129

[B3] Yoo I Song M Biomedical ontologies and text mining for biomedicine and Healthcare-A survey J Comput Sci Eng 2008 2 2 109 136 10.5626/JCSE.2008.2.2.109

[B4] Demner-Fushman D Antani S Simpson MS Thoma GR Design and development of a multimodal biomedical information retrieval system J Comput Sci Eng 2012 6 2 168 177 10.5626/JCSE.2012.6.2.168

[B5] Skounakis M Craven M Ray S Hierarchical hidden Markov models for information extraction Proceedings of the Eighteenth International Joint Conference on Artificial Intelligence 2003 San Francisco: Morgan Kaufmann Publishers Inc. 427 433

[B6] Chun H Tsuruoka Y Kim J Shiba R Nagata N Hishiki T Tsujii J Automatic recognition of topic-classified relations between prostate cancer and genes using MEDLINE abstracts BMC Bioinformatics 2006 7 Suppl 3 S4 10.1186/1471-2105-7-S3-S4 17134477 PMC1764448

[B7] Masseroli M Kilicoglu H Lang F Rindflesch T Argument-predicate distance as a filter for enhancing precision in extracting predications on the genetic etiology of disease BMC Bioinformatics 2006 7 291 10.1186/1471-2105-7-291 16762065 PMC1564420

[B8] Cheng D Knox C Young N Stothard P Damaraju S Wishart DS PolySearch: a web-based text mining system for extracting relationships between human diseases, genes, mutations, drugs and metabolites Nucleic Acids Res 2008 36 Suppl 2 W399 W405 18487273 10.1093/nar/gkn296 PMC2447794

[B9] Bundschus M Dejori M Stetter M Tresp V Kriegel H Extraction of semantic biomedical relations from text using conditional random fields BMC Bioinformatics 2008 9 207 10.1186/1471-2105-9-207 18433469 PMC2386138

[B10] Fang Y Lai P Dai H Hsu W MeInfoText 2.0: gene methylation and cancer relation extraction from biomedical literature BMC Bioinformatics 2011 12 471 10.1186/1471-2105-12-471 22168213 PMC3266364

[B11] Kim J Ohta T Tsujii J Corpus annotation for mining biomedical events from literature BMC Bioinformatics 2008 9 10 10.1186/1471-2105-9-10 18182099 PMC2267702

[B12] Friedman C Kra P Yu H Krauthammer M Rzhetsky A GENIES: a natural-language processing system for the extraction of molecular pathways from journal articles Bioinformatics 2001 17 suppl 1 S74 S82 10.1093/bioinformatics/17.suppl_1.S74 11472995

[B13] Pyysalo S Ohta T Miwa M Cho HC Tsujii J Ananiadou S Event extraction across multiple levels of biological organization Bioinformatics 2012 28 18 i575 i581 10.1093/bioinformatics/bts407 22962484 PMC3436834

[B14] Pyysalo S Ohta T Rak R Sullivan D Mao C Wang C Sobral B Tsujii J Ananiadou S Overview of the ID, EPI and REL tasks of BioNLP Shared Task 2011 BMC Bioinformatics 2012 13 Suppl 11 S2 10.1186/1471-2105-13-S11-S2 22759456 PMC3384257

[B15] Pyysalo S Ohta T Ananiadou S Overview of the Cancer Genetics (CG) task of BioNLP Shared Task 2013 Proceedings of the BioNLP Shared Task 2013 Workshop, ACL 2013 2013 Stroudsburg: Association for Computational Linguistics 58 66

[B16] Haber DA Settleman J Cancer: drivers and passengers Nature 2007 446 7132 145 146 10.1038/446145a 17344839

[B17] Craven Group Information Extraction Data Sets [ http://www.biostat.wisc.edu/~craven/ie/]

[B18] Gerner M Nenadic G Bergman CM An exploration of mining gene expression mentions and their anatomical locations from biomedical text Proceedings of the 2010 Workshop on Biomedical Natural Language Processing 2010 Stroudsburg: Association for Computational Linguistics 72 80

[B19] Verspoor K Jimeno Yepes A Cavedon L McIntosh T Herten-Crabb A Thomas Z Plazzer JP Annotating the biomedical literature for the human variome Database 2013 2013 10.1093/database/bat019, [ http://database.oxfordjournals.org/content/2013/bat019.full] 10.1093/database/bat019 PMC3676157 23584833

[B20] Amberger J Bocchini CA Scott AF Hamosh A McKusick’s Online Mendelian Inheritance in Man (OMIM) Nucleic Acids Res 2009 37 suppl 1 D793 D796 18842627 10.1093/nar/gkn665 PMC2686440

[B21] Garten Y Altman R Pharmspresso: a text mining tool for extraction of pharmacogenomic concepts and relationships from full text BMC Bioinformatics 2009 10 Suppl 2 S6 10.1186/1471-2105-10-S2-S6 19208194 PMC2646239

[B22] Thorn CF Klein TE Altman RB Pharmacogenomics and bioinformatics: PharmGKB Pharmacogenomics 2010 11 4 501 505 10.2217/pgs.10.15 20350130 PMC3098752

[B23] Müller HM Kenny EE Sternberg PW Textpresso: an ontology-based information retrieval and extraction system for biological literature PLoS Biol 2004 2 11 e309 10.1371/journal.pbio.0020309 15383839 PMC517822

[B24] Kim J So S Lee HJ Park JC Kim Jj Lee H DigSee: disease gene search engine with evidence sentences (version cancer) Nucleic Acids Res 2013 41 W1 W510 W517 10.1093/nar/gkt531 23761452 PMC3692119

[B25] Friedman C A broad-coverage natural language processing system Proceedings of the AMIA Symposium 2000 Richmond: American Medical Informatics Association 270 270 PMC2243979 11079887

[B26] Kim JD Pyysalo S Ohta T Bossy R Nguyen N Tsujii J Overview of bionlp shared task 2011 Proceedings of the BioNLP Shared Task 2011 Workshop 2011 Stroudsburg: Association for Computational Linguistics 1 6

[B27] Stenetorp P Pyysalo S Topić G Ohta T Ananiadou S Tsujii J BRAT: a web-based tool for NLP-assisted text annotation Proceedings of the Demonstrations at the 13th Conference of the European Chapter of the Association for Computational Linguistics, EACL ’12 2012 Stroudsburg: Association for Computational Linguistics 102 107

[B28] Hanahan D Weinberg RA The hallmarks of cancer Cell 2000 100 57 70 10.1016/S0092-8674(00)81683-9 10647931

[B29] Hanahan D Weinberg RA Hallmarks of cancer: the next generation Cell 2011 144 5 646 674 10.1016/j.cell.2011.02.013 21376230

[B30] Sleator R Walsh P An overview of in silico protein function prediction Arch Microbiol 2010 192 3 151 155 10.1007/s00203-010-0549-9 20127480

[B31] Magrane M Consortium U UniProt knowledgebase: a hub of integrated protein data Database 2011 2011 10.1093/database/bar009 PMC3070428 21447597

[B32] Maglott D Ostell J Pruitt KD Tatusova T Entrez gene: gene-centered information at NCBI Nucleic Acids Res 2011 39 suppl 1 D52 D57 21115458 10.1093/nar/gkq1237 PMC3013746

[B33] Zhao M Sun J Zhao Z TSGene: a web resource for tumor suppressor genes Nucleic Acids Res 2013 41 D1 D970 D976 10.1093/nar/gks937 23066107 PMC3531050

[B34] Vogelstein B Papadopoulos N Velculescu VE Zhou S Diaz LA Kinzler KW Cancer genome landscapes Science 2013 339 6127 1546 1558 10.1126/science.1235122 23539594 PMC3749880

[B35] Higgins ME Claremont M Major JE Sander C Lash AE CancerGenes: a gene selection resource for cancer genome projects Nucleic Acids Res 2007 35 suppl 1 D721 D726 17088289 10.1093/nar/gkl811 PMC1781153

[B36] Mishra A Verma M Cancer biomarkers: are we ready for the prime time? Cancers 2010 2 190 208 10.3390/cancers2010190 24281040 PMC3827599

[B37] Holley JW Guilford JP A note on the G index of agreementr Educ Psychol Meas 1964 24 4 749 753 10.1177/001316446402400402

[B38] Feinstein AR Cicchetti DV High agreement but low Kappa: I. the problems of two paradoxes J Clin Epidemiol 1990 43 6 543 549 10.1016/0895-4356(90)90158-L 2348207

[B39] Maqungo M Kaur M Kwofie SK Radovanovic A Schaefer U Schmeier S Oppon E Christoffels A Bajic VB DDPC: Dragon database of genes associated with prostate cancer Nucleic Acids Res 2011 39 Suppl 1 D980 D985 20880996 10.1093/nar/gkq849 PMC3013759

[B40] Bodenreider O The Unified Medical Language System (UMLS): integrating biomedical terminology Nucleic Acids Res 2004 32 Suppl 1 D267 D270 14681409 10.1093/nar/gkh061 PMC308795

[B41] Kao J Salari K Bocanegra M Choi Y Girard L Gandhi J Kwei KA Hernandez-Boussard T Wang P Gazdar AF Minna JD Pollack JR Molecular profiling of breast cancer cell lines defines relevant tumor models and provides a resource for cancer gene discovery PLoS ONE 2009 4 7 e6146 10.1371/journal.pone.0006146 19582160 PMC2702084

[B42] Tsuji K Kawauchi S Saito S Furuya T Ikemoto K Nakao M Yamamoto S Oka M Hirano T Sasaki K Breast cancer cell lines carry cell line-specific genomic alterations that are distinct from aberrations in breast cancer tissues: Comparison of the CGH profiles between cancer cell lines and primary cancer tissues BMC Cancer 2010 10 15 10.1186/1471-2407-10-15 20070913 PMC2836299

[B43] Lacroix M Leclercq G Relevance of breast cancer cell lines as models for breast tumours: an update Breast Cancer Res Treat 2004 83 3 249 289 10.1023/B:BREA.0000014042.54925.cc 14758095

[B44] Neve RM Chin K Fridlyand J Yeh J Baehner FL Fevr T Clark L Bayani N Coppe J Tong F Speed T Spellman PT DeVries S Lapuk A Wang NJ Kuo W Stilwell JL Pinkel D Albertson DG Waldman FM McCormick F Dickson RB Johnson MD Lippman M Ethier S Gazdar A Gray JW A collection of breast cancer cell lines for the study of functionally distinct cancer subtypes Cancer Cell 2006 10 6 515 527 10.1016/j.ccr.2006.10.008 17157791 PMC2730521

[B45] Lambros MB Fiegler H Jones A Gorman P Roylance RR Carter NP Tomlinson IP Analysis of ovarian cancer cell lines using array-based comparative genomic hybridization J Pathol 2005 205 29 40 10.1002/path.1681 15586366

[B46] Langdon SP Lawrie SS Establishment of ovarian cancer cell lines Ovarian Cancer, Volume 39. Edited by Bartlett JMS 2001 New York: Humana Press 155 159 doi:10.1385/1-59259-071-3:155 10.1385/1-59259-071-3:155 21340766

[B47] Kaur M Radovanovic A Essack M Schaefer U Maqungo M Kibler T Schmeier S Christoffels A Narasimhan K Choolani M Bajic VB Database for exploration of functional context of genes implicated in ovarian cancer Nucleic Acids Res 2009 37 Suppl 1 D820 D823 18790805 10.1093/nar/gkn593 PMC2686485

[B48] Langland GT Yannone SM Langland RA Nakao A Guan Y Long SBT Vonguyen L Chen DJ Gray JW Chen F Radiosensitivity profiles from a panel of ovarian cancer cell lines exhibiting genetic alterations in p53 and disparate DNA-dependent protein kinase activities Oncol Rep 2010 23 4 1021 1026 20204287 10.3892/or_00000728 PMC2909445

[B49] Sobel RE Sadar MD Cell lines used in prostate cancer research: a compendium of old and new lines - part 1 J Urol 2005 173 2 342 359 10.1097/01.ju.0000141580.30910.57 15643172

[B50] Sobel RE Sadar MD Cell lines used in prostate cancer research: a compendium of old and new lines - part 2 J Urol 2005 173 2 360 372 10.1097/01.ju.0000149989.01263.dc 15643173

[B51] The Prostate Cancer Cell Line Database [ http://capcelllines.ca]

[B52] Browne AC Divita G Aronson AR McCray AT UMLS language and vocabulary tools Proceedings of the AMIA Annual Symposium 2003 Richmond: American Medical Informatics Association 798 798 PMC1480270 14728303

[B53] Charniak E Johnson M Coarse-to-fine n-best parsing and MaxEnt discriminative reranking Proceedings of the 43rd ACL 2005 Stroudsburg: Association for Computational Linguistics 173 180

[B54] McClosky D Any domain parsing: automatic domain adaptation for natural language parsing. PhD thesis 2009 Brown University: Department of Computer Science

[B55] De Marneffe MC MacCartney B Manning CD Generating typed dependency parses from phrase structure parses Proceedings of the LREC 2006 2006 2006 [ http://www.lrec-conf.org/proceedings/lrec2006]

[B56] Leaman R Gonalez G BANNER: An executable survey of advances in biomedical named entity recognition Proceedings of the Pacific Symposium on Biocomputing 2008 Hackensack: World Scientific 652 663 18229723

[B57] Smith L Tanabe L Ando R Kuo C Chung I Hsu C Lin Y Klinger R Friedrich C Ganchev K Torii M Liu H Haddow B Struble C Povinelli R Vlachos A Baumgartner W Hunter L Carpenter B Tsai R Dai HJ Liu F Chen Y Sun C Katrenko S Adriaans P Blaschke C Torres R Neves M Nakov P Divoli A Mana-Lopez M Mata J Wilbur WJ Overview of BioCreative II gene mention recognition Genome Biol 2008 9 Suppl 2 S2 10.1186/gb-2008-9-s2-s2 18834493 PMC2559986

[B58] Björne J Ginter F Heimonen J Airola A Pahikkala T Salakoski T Extracting complex biological events with rich graph-based features sets Proceedings of the BioNLP’09 Shared Task on Event Extraction 2009 Stroudsburg: Association for Computational Linguistics 10 18

[B59] Kim JD Ohta T Pyysalo S Kano Y Tsujii J Overview of BioNLP’09 shared task on event extraction Proceedings of the Workshop on Current Trends in Biomedical Natural Language Processing: Shared Task, BioNLP ’09 2009 Association for Computational Linguistics 1 9

[B60] Aronson AR Effective mapping of biomedical text to the UMLS Metathesaurus: the MetaMap program Proceedings of the AMIA Symposium 2001 Richmond: American Medical Informatics Association 17 17 PMC2243666 11825149

[B61] Medlock B Briscoe T Weakly supervised learning for hedge classification in scientific literature ACL, Volume 2007 2007 Stroudsburg: Association for Computational Linguistics 992 999

[B62] Kilicoglu H Bergler S Recognizing speculative language in biomedical research articles: a linguistically motivated perspective BMC Bioinformatics 2008 9 Suppl 11 S10 10.1186/1471-2105-9-S11-S10 19025686 PMC2586760

