# Supplementary material for: CoMAGC: a corpus with multi-faceted annotations of gene-cancer relations
Source: BMC Bioinformatics. 2013 Nov 14;14:323. doi: 10.1186/1471-2105-14-323 (PMC3833657; doi:10.1186/1471-2105-14-323)
Supplement: Additional file 1 — Corpus annotation guidelines. A '.doc’ file that contains the definitions of annotation concept values, example annotations and instructions on allowed or disallowed types of inference during annotation. [file 1471-2105-14-323-S1.docx]

**Guidelines for**

**Multi-faceted Annotation of Gene-Cancer Relations**

Hee-Jin Lee^1^, Sang-Hyung Shim^2^, Mi-Ryoung Song^2^, Hyunju Lee^3^ and Jong C. Park^1^

^1^Department of Computer Science, KAIST

^2^School of Life Sciences, Bioimaging Research Center and Cell Dynamics Research Center, Gwangju Institute of Science and Technology

^3^School of Information and Communications, Gwangju Institute of Science and Technology

**1. Annotation examples**

In this section, we show annotation examples for each of the four annotation concepts. In the examples, an annotation unit is represented by marking the gene name, cancer-related terms and the keywords for gene expression change with square brackets and subscripts ‘g', ‘c' and ‘e', respectively.

**(1) Examples for CGE**

(Ex. 1)

Transfection-mediated [WWOX]_g_ [overexpression]_e_ in [DU145]_c_ cells suppressed colony growth (P = 0.0012). [PMID: 16818616]

🡺 CGE: increased

(Ex. 2)

[Down-regulation]_e_ of [ATM]_g_ protein sensitizes human [prostate cancer]_c_ cells to radiation-induced apoptosis. [PMID: 15837784]

🡺 CGE: decreased

**(2) Examples for CCS**

(Ex. 3)

We have found that [Cdc6]_g_ [expression]_e_ is [down-regulated]_e_ in [prostate cancer]_c_ as detected by semiquantitative reverse transcriptase-PCR of prostate cell lines and laser-captured microdissected prostate tissues. [PMID: 12006585]

🡺 CCS: normal->cancer

(Ex. 4)

[Increased]_e_ [expression]_e_ of [cyclin B1]_g_ sensitizes [prostate cancer]_g_ cells to apoptosis induced by chemotherapy. [PMID: 17513602]

🡺 CCS: cancer->normal; weakened cancerous properties.

(Ex. 5)

These results demonstrate that in a sequence-specific manner, RIalpha antisense, via efficient [depletion]_e_ of the [growth stimulatory molecule RIalpha]_g_, induces growth inhibition, apoptosis, and phenotypic (cell morphology) changes, providing an innovative approach to combat hormone-insensitive [prostate cancer]_c_ cell growth. [PMID: 11839683]

🡺 CCS: cancer->normal

(Ex. 6)

The insLQ polymorphism [increases]_e_ [LHR]_g_ activity , thereby shortening [breast cancer]_c_ disease-free survival, probably by increasing estrogen exposure in female carriers. [PMID: 16464948]

🡺 CCS: unidentifiable; we cannot infer the value of CCS from the information about patients survival (cf. Section 2).

(Ex. 7)

We conclude that [c-erbB-2 protein]_g_ [overexpression]_e_ assessed by IHC is a superior prognostic indicator in operable [breast cancer]_c_ compared to c-erbB-2 gene amplification analysed by the dot-blot technique. [PMID: 7917361]

🡺 CCS: normal->cancer; the information that one experimental technique is superior to another experimental technique is not relevant to the value of CCS.

(Ex. 8)

Collectively, the results of this study suggest that only the combined [inhibition]_e_ of [Cyclin D1]_g_ and D3 might be a suitable strategy for [breast cancer]_c_ prevention and therapy. [PMID: 22037875]

🡺 CCS: unidentifiable; the sentence does not contain the information whether the cancer progression would be prevented or not when only Cycline D1 is inhibited.

(Ex. 9)

No statistically significant association was found between [increased]_e_ [PTPase]_g_ activity and either c-erbB-2 overexpression or grade and stage of disease in primary human [mammary tumors]_c_. [PMID: 1349318]

🡺 CCS: unidentifiable; the information about gene-cancer relation is negated. We cannot infer the value of CCS when only negated information is provided.

(Ex. 10)

Southern analysis of [breast cancer]_c_ profiling arrays revealed that 29 patients (group I) expressed an elevated LF, 10 patients (group II) showed [decreased]_e_ [LF]_g_, and 8 patients (group III) had no change relative to the adjacent normal tissue. [PMID: 15313907]

🡺 CCS: normal->cancer; decreased LF accompanies progress of a certain type of breast cancer.

**(3) Examples for IGE**

(Ex. 11)

Transfection-mediated [WWOX]_g_ [overexpression]_e_ in [DU145]_c_ cells suppressed colony growth (P=0.0012). [PMID: 16818616]

🡺 IGE: unidentifiable

(Ex. 12)

Strikingly, IFN-gamma-induced apoptosis and growth inhibition of [M12]_c_ cells were associated with persistent [suppression]_e_ of the constitutive tyrosine-phosphorylated [STAT3]_g_ (pY-STAT3). [PMID: 16427044]

🡺 IGE: unidentifiable

(Ex. 13)

The gene array revealed [decreased]_e_ [expression]_e_ of ADAMTS1, [ephrin-A5]_g_, fibronectin 1, and neuropilin 1 in [LNCaP-19]_c_ compared to [LNCaP]_c_, while expression of midkine and VEGF were increased. [PMID: 18076023]

🡺 IGE: unidentifiable

**(4) Examples for PT**

(Ex. 14)

An [increase]_e_ in the activity of [mitogen-activated protein kinase]_g_ (MAPK) has been correlated with the progression of [prostate cancer]_c_ to advanced disease in humans. [PMID: 15833840]

🡺 PT: observation

(Ex. 15)

These observations suggest that U19 is growth inhibitory and tumor suppressive and that the disruption of androgen-dependent growth inhibition via [U19]_g_ [down-regulation]_e_ is commonly associated with [prostate cancer]_c_ progression. [PMID: 12907652]

🡺 PT: causality; the causality is inferred from the description ‘U19 is growth inhibitory and tumor suppressive’.

**2. Allowed or disallowed types of inference**

For the multi-faceted annotation in CoMAGC, the annotators are allowed to perform inference. Below are instructions on the spectrum of inferences that are allowed or disallowed during the annotation.

**① Annotators can interpret the sentences and annotate concepts in a ‘conventional way’, in which the sentences would usually be interpreted by human readers.**

(Ex. 16)

We have found that [Cdc6]_g_ [expression]_e_ is [down-regulated]_e_ in [prostate cancer]_c_ as detected by semiquantitative reverse transcriptase-PCR of prostate cell lines and laser-captured microdissected prostate tissues. [PMID: 12006585]

🡺 CCS: normal->cancer; although there is no explicit expression about the cell or tissue state change, we interpret the expression ‘down-regulated in prostate cancer’ as describing change in expression level of the gene relative to the normal state, and assign ‘normal->cancer’ to CCS.

(Ex. 17)

[Overexpression]_e_ of the enhancer of zeste homolog 2 ([EZH2]_g_) protein, a known repressor of gene transcription, has been reported to be associated with biological malignancy of [prostate cancer]_c_ and several other cancers. [PMID: 16734726]

🡺 CCS: normal->cancer; although the word ‘associated’ itself does not specify whether the gene overexpression stimulates or attenuates the malignancy of prostate cancer, we interpret the word as having the meaning of ‘positive regulation’ and assign ‘normal->cancer’ to CCS.

(Ex. 18)

These findings suggest that the quinazoline-based doxazosin mediates [prostate cancer]_c_ apoptosis by initially [inducing]_e_ the [expression]_e_ of [TGF-beta1]_g_ signalling effectors and subsequently I kappa B alpha. [PMID: 12771931]

🡺 CCS: cancer->normal; although the word ‘mediates’ itself does not specify whether the induction of gene expression stimulates or attenuates apoptosis, we interpret the word as having the meaning of `positive regulation’ and assign ‘cancer->normal’ to CCS.

**② Annotators can infer information using their prior knowledge about properties of cancer cells when the sentence is about comparison of two different cancer cells of the same cancer type.**

(Ex. 19)

Compared with [BPH]_c_, the [PCa]_c_ patients showed [decreased]_e_ [expressions]_e_ of [miR-98]_g_, let-7d and let-7g, and decreased expressions of miR-96, miR-182 and miR-183, with statistically significant differences between the two groups (P<0.05). [PMID: 20873592]

🡺 CCS: normal->cancer; from the prior knowledge that BPH is a benign cell while PCa is malignant, we can assign ‘normal->cancer’ to CCS.

(Ex. 20)

The gene array revealed [decreased]_e_ [expression]_e_ of [ADAMTS1]_g_, ephrin-A5, fibronectin 1, and neuropilin 1 in [LNCaP-19]_c_ compared to [LNCaP]_c_, while expression of midkine and VEGF were increased. [PMID: 18076023]

🡺 CCS: cancer->normal; from the prior knowledge that LNCaP-19 is androgen-independent and LNCaP is androgen-dependent, we can assign ‘cancer->normal’ to CCS. Androgen-independent cells do not respond to anti-androgen therapy while androgen-dependent cells do.

**③ Annotators can infer information utilizing linguistic clues.**

(Ex. 21)

Treatment of the androgen-independent human [prostate cancer]_c_ cells [PC-3]_c_ with doxazosin resulted in a strong [caspase-3]_g_ [activation]_e_ within 24h, whereas tamsulosin, a sulphonamide-based alpha 1-adrenoceptor antagonist, had no significant apoptotic effect against [prostate cancer]_c_ cells. [PMID: 12771931]

🡺 CCS: cancer->normal; although there is no explicit information about the effect of caspase-3 activation on prostate cancer cells, we can infer that it has ‘apoptotic effect against prostate cancer cells’ from the subordinate clause led by the word ‘whereas’.

**④ Annotators should not infer information using their prior knowledge about the functions of genes.**

(Ex. 22)

Elucidating the molecular events resulting from [loss]_e_ of [AP-2]_g_ in the prostate epithelium has implications for the understanding and prevention of the onset of [prostate cancer]_c_. [PMID: 14744478]

🡺 CCS: unidentifiable; prior knowledge about the function of AP-2 should not be used to identify the value of CCS. From only the information in the sentence above, we cannot identify whether the loss of AP-2 accompanies change in cancerous properties or not.

**⑤ Annotators should not infer the CCS value from the information about patients’ survival rates because progression of cancer cells or tissues is not the sole factor that contributes to patient survival or death.**

(Ex. 23)

The insLQ polymorphism [increases]_e_ [LHR]_g_ activity , thereby shortening [breast cancer]_c_ disease-free survival, probably by increasing estrogen exposure in female carriers. [PMID: 16464948]

🡺 CCS: unidentifiable

**⑥ Annotators need not consider the certainty level of propositions.**

(Ex. 24)

We conclude that 13q34 amplification may be of relevance in tumor progression of basal-like [breast cancers]_c_ by inducing [overexpression]_e_ of [CUL4A]_g_ and TFDP1, which are both important in cell cycle regulation. [PMID: 19995430]

🡺 CCS: normal->cancer; although the phrase ‘may be of relevance’ expresses uncertainty, we can ignore the uncertainty and assign ‘normal->cancer’ to CCS.
